# Early experiences of the End of Life Choice Act 2019 amongst assisted dying practitioners in Aotearoa New Zealand

**DOI:** 10.1186/s12904-025-01747-w

**Published:** 2025-05-24

**Authors:** Aida Dehkhoda, Rosemary Frey, Melissa Carey, Jacqualine Robinson, Frederick Sundram, Nicholas Hoeh, Susan Bull, Gary Cheung

**Affiliations:** 1 University of Auckland, Auckland, New Zealand; 2 University of Southern Queensland, Queensland, Australia; 3 University of Oxford, Oxford, England

**Keywords:** Assisted dying, Practitioners, Early experience, The Act, New Zealand

## Abstract

**Background:**

The global trend of legalising assisted dying (AD) has reshaped end-of-life care practices, and Aotearoa New Zealand’s adoption of the End of Life Choice Act (the Act) in 2019 represents a significant shift. Limited empirical research on AD in New Zealand after the enactment of the Act underscores the need for investigation. Conducting research in the early stages of AD implementation is crucial to building a strong knowledge base and laying the foundation for future research. This would ensure equitable and suitable service provision for the service users.

**Aim:**

This research captured the experiences of health practitioners directly involved in providing AD under New Zealand’s End of Life Choice Act 2019.

**Design:**

Using the “memorable case” approach, 22 participants reflected on the process of assessing, treating, and delivering AD services in the first 12 months of implementing the new AD law.

**Results:**

Thematic analysis identified four major themes underlying the experiences of assisted dying practitioners/providers (ADPs). The themes focused on three aspects of ADPs’ experience: KNOWING: prior personal experience (personal beliefs, clinical background, and AD training) and reflective experiences of DOING assessments, service delivery, and patient/family experiences and BEING an ADP (personal, professional, emotional, and social impacts). Additionally, the themes highlighted the overarching influence of health system infrastructure, challenges, and resources that shaped ADPs’ overall experience.

**Conclusion:**

These findings contribute to new knowledge by uncovering gaps in understanding, competency, service implementation, and the emotional impact on ADPs. The findings could inform the development of an educational, supportive, and culturally safe program, including resources for workforce development.

**Supplementary Information:**

The online version contains supplementary material available at 10.1186/s12904-025-01747-w.

## Introduction

Over the past two decades, the legalisation of assisted dying (AD) in many countries has introduced a notable change in end-of-life clinical practice. In Aotearoa New Zealand the End of Life Choice Act 2019 (the Act) came into force in 2021 [1]. The Act enables competent New Zealand citizens or permanent residents aged over 18 years with a terminal illness and a prognosis of less than six months, to apply for an assisted death. Applicants must be in an advanced state of irreversible physical decline and experiencing unbearable suffering that cannot be relieved in a manner that they consider tolerable [2].

Applications for AD are subject to independent assessments by two medical practitioners, an Attending Medical Practitioner (AMP) and an Independent Medical Practitioner (IMP). In addition, if concerns are raised by the AMP or IMP in relation to a person’s level of competency, a competency assessment undertaken by a psychiatrist is required. An attending Nurse Practitioner (ANP) who works with the patient’s AMP, can also be involved in the planning of AD and the administration of medicines or the supervision of self-administration. The AD medicines are provided on prescription only directly to practitioners delivering the services. It is delivered through two Health New Zealand registered pharmacies in a medication kit that contains all other necessary items to carry out the procedure, including a step-by-step guide. The Act’s safeguards also include a prohibition on health professionals from initiating conversations about AD with patients. In addition, the Act does not allow people who are deemed to be incompetent to access AD, nor does it allow for competent individuals to access AD far in the future by using advance directives should they become incompetent [1]. The New Zealand Ministry of Health [MoH] AD Registrar oversees the assessment process to ensure it meets the legislation requirements. A team of clinical nurse advisors supports the process, serving as the main patient and AD provider contact [3].

New assisted dying practitioners (ADPs) who have limited training or experience in AD face many new clinical responsibilities [4–6]. There are also potential personal and professional implications for ADPs, including ethical, existential, emotional, and logistical challenges [7–9]. The newly established AD legal framework in New Zealand and the inherent ambiguity within the eligibility criteria outlined in the Act have already raised some concerns for healthcare practitioners and patients [10]. For example, there is ambiguity around the meaning of terminal illness and unbearable suffering and assessment of 6-month life expectancy.

There is a very limited empirical investigation into AD in New Zealand since the Act came into force [11]. International research highlights the diversity of communication, support, professionalism, and information needs required throughout the AD process [8, 12–18]. It is, therefore, important that research is undertaken during the early introduction of AD to ensure that the service is equitable and appropriate for the population it is designed to serve.

This study aims to explore the early experiences of ADPs in New Zealand. By doing so, we will understand how AD services are being implemented and incorporated into the broader landscape of end-of-life care, build a knowledge base, and lays the foundation for future research. This timely and local research is essential for identifying challenges and concerns about the safety and accessibility of AD services within the unique culturally diverse context of New Zealand [19] with a mix of First Nations [Māori], European, Pacific Islander, Asian, and other ethnic groups. This proactive approach of exploring early phases and experiences will provide valuable insights to address these concerns and ensure that regulation reforms are informed by evidence and can be adjusted to better serve the community’s needs. It can ultimately contribute to the AD service that is safe, equitable, and effective [20] and to the limited local body of knowledge about AD in New Zealand.

## Methods

This study used an exploratory, qualitative design with an interpretative phenomenological approach [21]. This approach allowed us to understand the common lived experiences of ADPs in New Zealand. Using this approach, we invited ADPs to openly reflect on their experiences assessing the eligibility criteria and delivering AD services.

### Participants

We used a combination of purposeful and snowballing sampling to recruit participants using the MoH’s AD training and communication email list. We also sent communications regarding the study through the (i) Australian and New Zealand Society of Palliative Medicine, (ii) New Zealand’s Assisted Dying Research Network, and (iii) Women in Medicine Facebook Group. A practitioner was eligible if they had been directly involved in providing AD either as an AMP, IMP or ANP. We recruited and interviewed study participants between June and September 2022, which fell within the first 12 months of AD being legally available in New Zealand.

### Semi-structured interviews

Informed by the Act and contemporary AD literature, a research team developed an interview guide. The team has expertise in psychology, sociology, gerontology, psychiatry, nursing, ethics, and Kaupapa Māori research. In addition, the Te Ārai Palliative Care Research Group provided Māori cultural advice on the study. The interview guide (see Appendix) initially starts with questions focusing on gathering basic and factual information and becomes gradually deeper and more personal and reflective as the interview progresses. It contains open-ended, probing questions to generate rich and in-depth answers that reflect participants’ experiences and stories [22] on topics such as assessment of eligibility criteria, administration of AD medication, resources, AD personal and professional impacts, and self-care.

A.D. conducted all semi-structured interviews. Data saturation was reached following the 22nd interview [23]. Interviews were conducted on the Zoom platform (*n* = 21) or in person (*n* = 1) and varied from 35 to 95 min in duration. All interviews were audio recorded with permission, de-identified, and transcribed verbatim by a professional transcriber who had signed a confidentiality agreement. Regular research meetings with the principal investigators and some of the team members were held to discuss the interviews and the generated content to adjust the guide when necessary.

### Data analysis

**Fig. 1:**
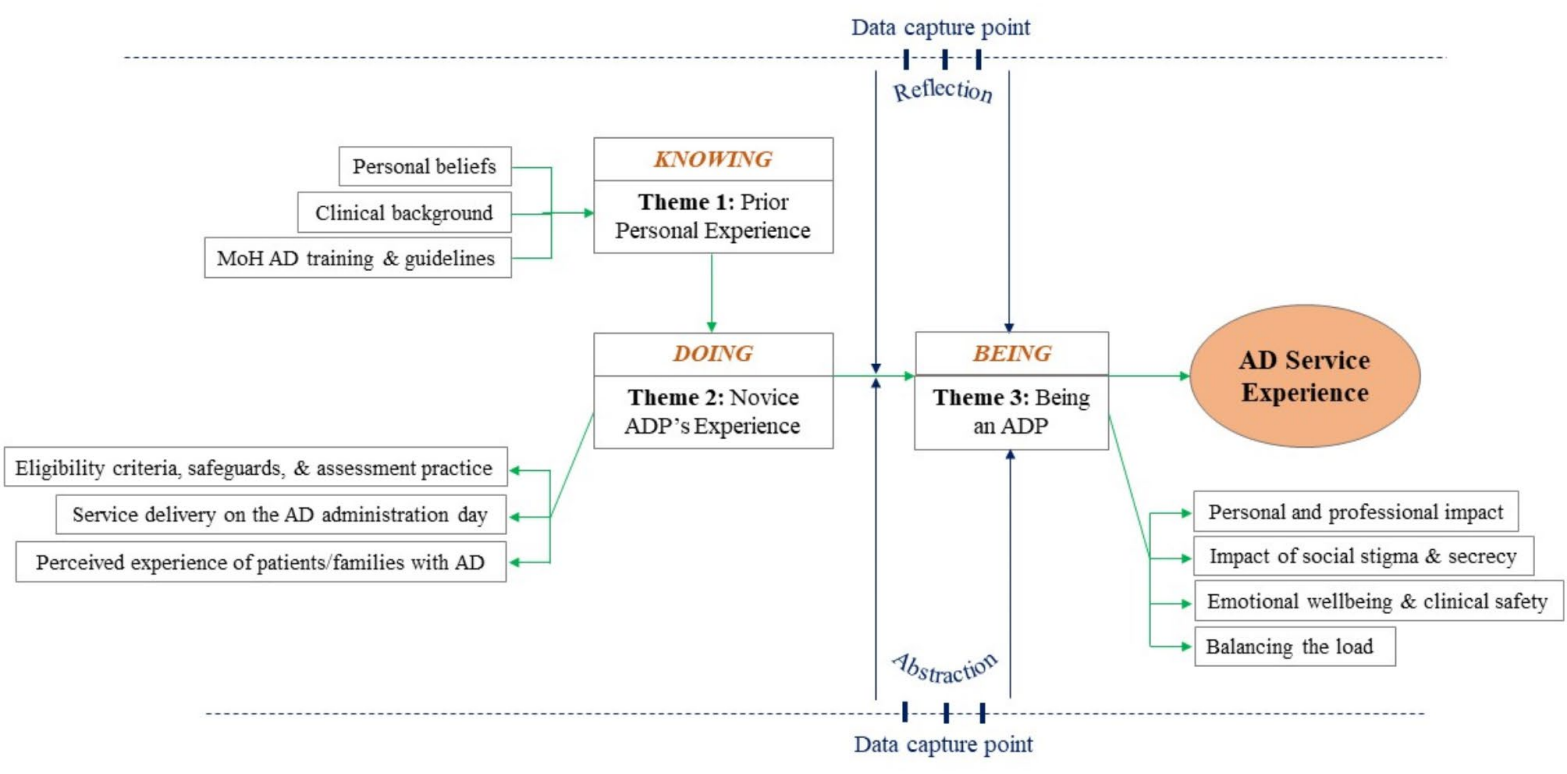
Thematic Diagram: Assisted dying service experience. AD = assisted dying; ADP = assisted dying practitioner; MoH = Ministry of Health

Transcripts were imported into NVivo (Release 1.5.2; QSR International). A process of reflexive thematic analysis [24, 25] was used to analyse the interview data. A.D. led the analysis in collaboration with M.C. and, after familiarising herself with the data, created a mind map based on the interview guide, which was used to create top-level codes. Working collaboratively, A.D. and M.C. further developed and refined the initial codes by employing an inductive approach. This involved extracting a range of analytic codes through careful reading of the data. The conceptual meaning of each code was discussed, interviews were coded line by line, and new sub/child codes were created iteratively by them for interview one and half of interview two. A.D. coded the rest of the data using the same technique, resulting in ten top-level codes and fifty-six child codes. Once this first coding round was finalised, A.D. and G.C. co-created the overarching themes by grouping the relevant codes. A thematic diagram (Fig. 1) was also developed at this stage with help from co-authors to visualise the conceptual interactions amongst the themes. This iterative process sometimes resulted in revisiting and adjusting the coding and mapping of themes until a consensus was reached. The outcome of this coding and analysis process yielded three primary themes and ten sub-themes, which are depicted in Fig. 1; Table 1 and presented below.

**Table 1:** Themes and sub-themes

Themes		Sub-themes
**Theme 1** **KNOWING–** Prior personal experiences		**1.1.** Personal beliefs **1.2.** Clinical background **1.3.** MoH training and guidelines
**Theme 2 DOING–** Early ADPs’ experience	**2.1.** Eligibility criteria, safeguards, and assessment practice	**2.1.1.** Perception of strictness and challenges in assessing eligibility criteria: • Terminal diagnosis and prognosis of less than six months • Competency and final consent • Unbearable suffering **2.1.2.** Perception of safeguards as barriers • Conscientious objection • Regulatory/statutory prohibition on raising AD **2.1.3.** Prior relationship with patients **2.1.4.** The importance of expertise
	**2.2.** Service delivery on the AD administration day	**2.2.1.** Medication kit and delivery methods **2.2.2.** The performance of AD and the final moments
	**2.3.** Perceived experience of patients and families with AD	**2.3.1.** End-of-life & AD literacy **2.3.2.** Perceived impact of AD on patients and families **2.3.3.** Cultural safety & ADP’s experience with Māori who applied for AD **2.3.4.** Ensuring safety and family support
**Theme 3** **BEING–** Being an ADP		**3.1.** Personal and professional impact of AD provision on ADPs **3.2.** Impact of social stigma and secrecy **3.3.** Emotional wellbeing and clinical safety of ADPs **3.4.** Balancing the load

AD = assisted dying; ADP = assisted dying practitioner; MoH = Ministry of Health

### Ethical considerations

This study received ethics approval from the University of Auckland Human Participants Ethics Committee (UAHPEC) (Ref. UAHPEC24335). Written informed consent was obtained from all participants. To ensure patient confidentiality, participants were instructed not to disclose identifying information about their patients and to use gender-neutral pronouns during the interviews. Trustworthiness was addressed by offering participants the opportunity to review the written transcriptions of their interviews, enabling them to verify the accuracy of reposting their views and make any desired revisions. A.D. removed all remaining identifying details and replaced names with numerical codes before importing the transcripts into NVivo for analysis. Moreover, we obtained consent from participants whose direct quotes were selected for publication. Pseudonyms have been utilised for exemplary quotes to protect participant identities.

## Findings

### Characteristics of participants

Twenty-two ADPs participated in the study, with a combination of attending and/or independent medical practitioners and one attending nurse practitioner (see Table 2). Although participants came from a variety of clinical backgrounds, most were general practitioners (68.1%). The details of their clinical backgrounds are not reported here to protect anonymity. At the time of the interviews, participants had been involved in approximately 230 AD assessments ranging from 3 to 30 cases for each participant.

**Table 2:** Demographic information of assisted dying practitioners (*n* = 22) who participated in this study

Demographic characteristic	Number (%) of participants *n* = 22		
**Gender**			
Male	11	(50)	
Female	11	(50)	
**Age**			
30–39	5	(22.7)	
40–49	7	(31.8)	
50–59	9	(40.9)	
> 60	1	(4.5)	
**Ethnicity**			
New Zealand European– Other European	16	(72.7)	
Asian	5	(22.7)	
Rather not say	1	(4.5)	
**Years of experience as a clinician**			
5–9	2	(9.1)	
10–19	7	(31.8)	
20–29	10	(45.4)	
> 30	3	(13.6)	
**Location**			
North Island	17	(77.2)	
South Island	5	(22.7)	
**Primary work setting**			
General Practice	15	(68.1)	
Hospital	7	(31.8)	
**Assisted dying role**			
Attending medical practitioner (AMP)	8	(36.3)	
Attending + independent medical practitioner (AMP + IMP)	13	(59.1)	
Attending nurse practitioner (ANP)	1	(4.5)	
**Completion of Ministry of Health e-learning modules** **For health professionals**			
End of Life Choice Act 2019: Overview	21	(95.4)	
Assisted dying care pathway: Overview	21	(95.4)	
Responding when a person raises assisted dying	21	(95.4)	
**For medical and nurse practitioners**			
Assessment process	20	(90.9)	
Preparation for an assisted death	20	(90.9)	
Assisted death and after-care	20	(90.9)	
Practitioner safety and wellbeing	18	(81.8)	
Module for review learning	18	(81.8)	

### Thematic framework

Despite the growing body of research on AD, there remains a notable lack of theoretical frameworks guiding this field [26]. Incorporating theoretical framework provides a structured lens helping to contextualise and interpret empirical data [27] and offer deeper insights into the complex ethical, legal, and social dimensions of AD. In this research, we used Heidegger’s philosophical concepts of knowing, experience, and practice [28, 29] as a thematic/theoretical framework. In interpreting the data through this lens, three overarching themes emerged which represent ADPs’ experiences in the first 12 months of providing an AD service in New Zealand. “Knowing,” akin to Heidegger’s holistic understanding, captures both theoretical and existential knowledge related to the complexity of AD. This theme pertains to ADP’s prior personal experience as contributing factors to their involvement in AD. The theme of “Doing” corresponds to the practical aspects of ADPs’ work, reflecting Heidegger’s notion of “Practice.” This delves into how ADPs’ actions in providing AD are linked to their ways of being and acting in the world, influenced by societal norms, values, and their own sense of being. “Being” parallels Heidegger’s existential focus, exploring how ADPs’ roles shape and are shaped by their lived experiences and emotional dimensions, contributing to a nuanced understanding of the intertwined nature of knowing, doing, and being in the realm of AD. These three themes and their sub-themes shape the overall AD service experience of ADPs (see Fig. 1). This thematic framework is further incorporated into the discussion. The description of themes follows.

### Theme 1: KNOWING – Prior personal experience

#### Personal beliefs: “I was brought up by a grandmother who strongly, strongly believed this should be available”

Several study participants expressed personal motivations for becoming an ADP. These motivations varied among participants, with some driven by long-standing beliefs in and advocacy for personal choice and bodily autonomy. For others, the decision to enter this practice was influenced by witnessing a relative or patient endure a protracted and agonising death. This evoked a sense of fear at the inability to alleviate the suffering.

A common underlying theme among some practitioners was the recognition that AD represents a choice they would desire for themselves, their loved ones, and their patients should they express such a wish. As a result, these individuals considered it morally important to support and enable this choice, which aligned with their fundamental values and beliefs. A participant shared:

Marcus: My [close relative] died of cancer, and that was a little bit of a battle. […]. Several years later, my other [close relative] got sick, and she did AD. And I found it such a good process, that somebody had a choice. And that they didn’t have to wait until the last moment because most of the people were dying. In the end, it’s a waiting game. So, I thought, okay, if somebody chooses not to go through that process [waiting to die without AD], it’s amazing that somebody else can help them with that. I’m also religious, so, of course, I had my what is good and what is not good. But finally, I said, hey, God has given me a talent, and I have this talent, and I try to use it for the good.

#### Clinical background: “I have helped people to die in my normal job anyway”

Clinical specialisations and practice settings influenced the desire to become an ADP for some participants. Practitioners working in fields such as abortion surgery, respiratory specialities, and intensive care units were more comfortable with the concept of death. Furthermore, the practice settings of some participants meant they had observed significant suffering in dying patients:

Chris: I was very against the concept. And, then I started working in the hospital and seeing people die and seeing people not die well. In some cases die particularly poorly, just suffer all the way until their soul left their body, and that really turned, changed my mind about it. Because I realised that’s, people die, people can die horribly. We have the ability to look after people and allow them to die peacefully, that’s something that we should be doing for people. But, it took me a while to come to terms with that, and it took practising medicine and experiencing that to understand it.

Notably, some practitioners believed there was a shortage of ADPs, especially in geographical areas already facing challenges in accessing healthcare. This scarcity played a role in determining their decision to provide AD services.

Maple: I had originally thought that I wanted to provide the service for just me and the patients that I look after as a general practitioner. And then the law went through and […] I decided to put my name on the SCENZ [Support and Consultation for End of Life in New Zealand Group] list to kind of have more support, I guess. And […] I had my first case. And it was around that time that I realised that I was probably the only AMP in the region that I work in. And so then I was like, oh, if there’s nobody else providing the service, then, you know, I really wanted to provide the service for the people that requested it.

#### Ministry of Health training and guidelines: “I think it’s important that we remain engaged with the continuing medical education on this [AD]”

All participants employed various methods to acquire the necessary knowledge and competency for providing AD within the New Zealand context. These included engaging in self-directed learning through activities such as listening to podcasts, reading books and journal articles, as well as attending workshops and courses focused on topics such as competency assessment and communication skills. Additionally, although not mandatory, most participants had completed the online e-learning modules developed by the MoH (see Table 2). Participants also described utilising guidelines and resources available on the MoH website to gather information. However, they noted that these resources were not always detailed enough to fully prepare ADPs for the assessment and delivery process, as outlined in the following sections.

Greg: There should be a module with all the pharmaceutical and medicinal and technical things. I was shocked to find that I had started an AMP assessment with no idea what medications we were using and what doses and how to give them.

### Theme 2: DOING – Early ADPs’ experience

#### Eligibility criteria, safeguards, and assessment practice

##### Perception of strictness and challenges in assessing eligibility criteria: “It [AD process] doesn’t really trust the patient to make the right decision”

ADPs regarded the regulatory safeguards and eligibility criteria as important to ensure the safety of both practitioners and patients in the AD context. However, there was a prevailing sentiment among participants that the criteria are excessively stringent and conservative, limiting accessibility for some patients. Many participants expressed frustration over the burdensome administrative processes and paperwork involved in the assessment process, perceiving them as unnecessary obstacles for patients seeking AD. One noted:

Evan: I feel like the assisted dying legislation was written for politicians rather than for dying patients. The patient’s voice in all of this is as a bystander who has to get dragged through dozens of pages of repetitive questions, multiple medical interviews, […], and proving to others that you’ve got ‘intolerable’ suffering. Those are a lot of hoops for sick patients to jump through.

*Terminal diagnosis and prognosis of less than six months*: Participants unanimously identified the requirement of having less than a six-month life expectancy as the most challenging criterion in practice for a variety of reasons. It was perceived to be nearly impossible to accurately predict life expectancy due to the unpredictable nature of many medical conditions, especially when a rapid and unexpected decline occurs– a not uncommon occurrence. In addition, it was also stated that some terminal and progressive conditions, like neurodegenerative diseases, do not fit neatly within a six-month timeframe but still lead to irreversible decline. As a result, this criterion excludes patients who would otherwise meet the eligibility requirements for AD.

John: The six-month criterion is very helpful if a well-known diagnosis is made for someone with pancreatic cancer. You know, it’s probably within that timeframe. However, certain things are much more, are much harder so to speak. For instance, I’ve got someone with motor neurone disease for example, and we know is a progressive disease, and most people don’t do very well. You know, probably about 18 months after their diagnosis, they tend to go downhill very quickly. However, that prognosis depends on what the patient does to his own health. Also, whether he would want to accept some of the supportive treatment or not. So, that makes it a lot more difficult to say whether they fit the criteria or not.

*Competency and final consent*: Most participants reported that assessing competency for AD is a relatively straightforward process. Their extensive prior clinical practice and experience in evaluating patients’ decision-making capacity contributed to their confidence in this area. Notably, most AD competency assessments were “clear-cut,” where patients were either deemed competent or not, and this pattern of clear-cut assessments extended to evaluating patients’ competency on the day of AD administration. Most participants observed that patients exhibited unwavering certainty regarding their wish for an assisted death on the day of administration. Furthermore, as the ADPs had developed a deeper relationship with the patients as well as knowledge of their medical conditions by that stage, they felt more confident in making judgments regarding patients’ capacity to consent.

Brianna: I think that’s called experience. […]. We [GPs] do it [capacity assessment] every minute of every day with every medication, with every intervention that we have. We just don’t call it something formal.

A theme that emerged from participants’ perspectives on competency, revolved around situations where patients, although no longer considered competent, had expressed their desire for an assisted death clearly and consistently. While this raised concerns among participants regarding the logistics and procedures of providing AD in such cases, their overall inclination leaned towards supporting the availability of this option in the future if appropriate safeguards, such as advance directives, were to be in place in the future. Indeed, many participants questioned the necessity of obtaining consent again on the day of AD administration. While some participants viewed the need for consent at the time of administering an AD as reassuring and affirming, others voiced concerns about patients who might lose capacity, for example through delirium. Some participants argued that denying access to AD in such situations would be unfair, as there is no reason to believe these patients would have changed their minds. Moreover, a requirement for final consent could potentially put patients at risk of harm if they were deemed to lack capacity and ineligible for AD.

Andre: One of the things we hear, not infrequently, is that I’m not going to take palliative care medication in case I become incompetent and can’t have the assisted death that I want. So, I’m prepared to increase my suffering, which is insane, so I can die when I want to.

Participants reported employing a holistic approach when conducting capacity assessments. They would typically evaluate whether patients demonstrated an understanding of AD and the various treatment options available to them. In addition, participants described how they assessed whether the patient could weigh the benefits and risks of each option in relation to their specific condition as well as retained and recalled relevant information from discussions and communicated their reasoning and decisions to the AMP and IMP.

Participants also emphasised to patients that they retained the autonomy to delay or halt the AD process at any stage. The administration and assessment process often takes several weeks, and in certain instances, participants reported that patients’ communication or verbal abilities deteriorated between assessment sessions. Despite the challenges posed by these situations, participants described how they relied on non-verbal forms of communication such as eye contact, nodding, and other cues to ascertain patients’ agreement or dissent in the absence of verbal confirmation. Participants reported that they would involve a psychiatrist for a more thorough evaluation of competency if competency is in doubt after their assessment, although such instances were infrequent.

Maple: I think that the involvement of psychiatrists is very limited at this stage from what I’ve seen.

Chris: my first IMP case was a very challenging assessment of someone who had a rapidly progressing brain tumour that was slowly eroding and affecting their personality and their motivation. Because, I hadn’t met them before, and I was just stepping there as, like, and you get, sort of, a snapshot of this person at this time and place that was, I found that very challenging. I mean, we’ve got the psychiatrist there to help back us up if we’re really worried. That’s a bit of a safety net and if you are umming and thing then that’s where you should go.

*Unbearable suffering*: Some participants reported that “unbearable suffering” is not clinically measurable. The subjective nature of evaluating the degree of unbearableness often results in confusion and poses challenges. Some participants commonly found themselves explaining and clarifying this criterion to patients, helping them to understand it and its implications. However, this subjectivity also allowed some room for participants to consider other non-physical or pain-related suffering such as emotional, psychological, and existential suffering. Some participants found the assessment of “unbearable suffering” straightforward, considering it to be entirely guided by the patient’s feelings and views. With this broader perspective on suffering, participants acknowledged the influence of the medical condition on various aspects of the patient’s life; such as social interactions, daily activities, relationships, autonomy, independence, plans, and overall quality of life. Some participants also identified the fear of losing control and dependence on others as a contributing factor to the experience of suffering:

Leyla: I have found that the fear of losing control is a huge factor in this. So many of the people that I’ve worked with, their main concern has been that they have either been people who have been in excellent control of their lives. So, they’ve been bankers, lawyers and accountants, and they’ve always had a degree of control over their lives. And they find it terrifying to be not in control. And so that causes a great deal of anxiety and suffering in people.

##### Perception of safeguards as barriers: “I have got first-hand experience of patients being stonewalled [due to conscientious objection]”

*Conscientious objection*: Participants consistently voiced concerns regarding the impact of conscientious objection on the accessibility and quality of AD services. They unanimously perceived conscientious objection as a significant barrier preventing individuals from accessing AD in a timely manner. Based on the participants’ experience and information shared with them, they reasoned how conscientious objection could lead to suboptimal AD provision at various levels.

On an individual level, some healthcare providers, including GPs, nurses, oncologists, and palliative care specialists/nurses, whose personal and professional values conflicted with AD, were identified by patients as withholding information about AD. Patients described experiences of healthcare professionals refusing to make referrals or provide the necessary information to access AD. This resulted in delaying the assessment process by withholding essential diagnostic and prognostic information when approached by ADPs seeking the information they needed.

There were also reports to participants of patients expressing anxiety about disclosing their desire for AD to their doctors, some known to be conscientious objectors, fearing strain on their doctor-patient relationships. At a systemic level, participants expressed frustration with the reluctance of most regulatory bodies, such as hospitals, hospices, aged residential care facilities, and rest homes, to allow AD assessment and/or provision on their premises. ADPs believed that the lack of support from these organisations and their staff had resulted in limited delivery sites, ultimately complicating or even making the AD service inaccessible:

Brianna: They’ve [in a hospital] put the room aside; it’s called the room of last resort. I mean, you can just tell from the language. I have a colleague about to do an assisted dying in the hospital. And the hoops they had to do, the people, people stopped them in the hallway and said, you know, I think you’re wrong. The nurses said if you come onto our ward, we won’t help you in any way! You’ll be left in that room! You know, it’s been very unpleasant.

*Regulatory/statutory prohibition on raising AD*: Many participants discussed challenges with the restrictions on initiating discussions about AD and informing patients about this end-of-life care option. They believed that having this safeguard in place potentially limits the provision of AD for two reasons. First, some participants had encountered patients whose illness was too advanced by the time they considered AD as an option. Second, withholding information about the full range of end-of-life care options ethically challenged some participants because they believed it denied patients the opportunity to make an appropriately informed decision. These ADPs argued that providing information about an option does not necessarily lead to medical coercion, in which patients are potentially feeling pressured to choose AD. On the other hand, smaller number of participants did not see this safeguard as a barrier. They believed that it prevents AD from being suggested as a treatment option and unintentionally influencing patients to view it as their best choice. However, many of these participants acknowledged the risk that some patients, particularly those in rural areas, may not be aware of AD.

##### Prior relationship with patients: “You get to judge them [patients’ clinical situation] and listen to them on their terms with no baggage brought with you”

Most participants generally had no prior relationship with the patients they assessed or assisted with AD and were ambivalent about the advantages and disadvantages of not having prior knowledge of the patients. Some participants highlighted the potential benefits of approaching the assessment and decision-making process with objectivity and independence, avoiding emotional attachment, and experiencing less intense grief reactions. On the other hand, the most common drawbacks mentioned by participants included challenges in establishing rapport and gaining patients’ trust within a limited number of sessions, understanding the progression of the disease and prognosis, and understanding the dynamics of the patient’s family and its influence on the AD request. Nonetheless, some participants felt that this did not significantly affect the assessment and provision of services, especially for IMPs who were in place to provide a second opinion rather than having a more involved role with patients and families.

Mattew: It doesn’t, I don’t think it’s made a difference with the relationship. It sometimes made a little bit of a difference with knowing their trajectory and, you know, if they’ve not, I guess, if they’ve not got such a clear cut.

##### The importance of expertise: “You need all of your six senses on just to detect whether there is something unusual going on.”

As participants took on more new cases, they perceived that providing AD necessitates a significant level of expertise and experience. Participants acknowledged that they relied primarily on their pre-existing competencies. These included interviewing and communication skills, managing complex emotions of patients and families, addressing physical decline at the terminal stage of their disease and intolerable suffering, establishing decision-making competency and obtaining informed consent, and demonstrating empathy throughout the entire process. Participants described how they would integrate their established medical competencies and AD knowledge with the experiential insight gained from each AD to inform and enhance their subsequent assessments and provisions. However, despite possessing clinical expertise, some participants faced challenges during the assessment process, particularly in cases where family members were made the main point of contact by the patient. In these cases, some participants found it difficult to determine the AD request’s authenticity and complete the eligibility criteria assessment.

In some instances, the two independent medical assessments regarding the patient’s eligibility conflicted; therefore, the patient was deemed ineligible for an AD. Participants described concerns that these patients were at risk of suicide if found ineligible. The participants faced ethical and psychological dilemmas when conveying the news of ineligibility to these patients, particularly those who were clearly suffering.

Considering all these challenges, some participants expressed concerns about inexperienced practitioners providing AD services, as it posed potential risks of undue harm to themselves and the patients involved.

Arianna: But I definitely don’t think these assessments should be done by junior doctors. You know, I’ve found that I’m using all of my skills. The communication skills, and, well, everything: the competency assessment and dealing with people. It’s not for juniors, really. Or shouldn’t be.

#### Service delivery on the AD administration day

##### Medication kit and delivery methods: “There’s a lot of stuff in there. It can be a bit intimidating”

Most participants expressed satisfaction with the AD medication kit, highlighting its clear instructions and comprehensive preparation. However, several ADPs identified some key issues in relation to the AD kit, such as missing gloves and tourniquets, and blood sheets not being the best quality compared to what they use in their practice, and which they considered essential. Additionally, participants raised concerns about the perceived wastefulness of the kit, as any unused items might be discarded. The size of the medication kit was also a point of conversation, with some participants finding it large and intimidating for patients and families. As a result, some opted to carry only the necessary items in a separate box, leaving the remaining components somewhere close by, for example, in their vehicles, as a backup. While the dispensing process for the AD kit was generally efficient, participants noted that the delivery of the medication kit could take up to a week. This was mostly for ADPs situated outside of Auckland, where one of the two pharmacies that dispatch AD medication is located. This delay posed risks in situations when AD provision needed to be expedited for any reason, such as a rapid decline in health.

Most participants, including those who had extensive clinical experience and completed AD learning modules and preparation, expressed a profound sense of unpreparedness when faced with their first assisted death. They described a lack of knowledge regarding the workings of the medication as well as administration challenges. Notably, participants who had experience with both oral and intravenous (IV) medications found a difference between the two methods, which caught them off guard:

Greg: I find that oral medication is very unsettling, and it is very difficult for families and patients. It is very upsetting to everyone watching the patient gagging and choking on this [medication name]. It’s really hard to watch. I wasn’t prepared for that the first time it happened […] and even the family as they were watching were saying, you know, his body’s trying to get rid of this stuff. […]. Whereas intravenous medication is a very quiet, peaceful, gentle way to go. […]. And it’s [oral medication] the longest kind of 15 to 30 min of your life watching someone as they’re struggling to breathe as the medication takes hold. With the intravenous, you’re looking at five minutes.

Some participants reported that even though they had perceived the IV medication to be more effective than the oral route, it was not without its drawbacks. Most participants encountered difficulties related to IV access due to factors, which they described as “burnt or dehydrated veins” due to prior chemotherapy treatments, swelling caused by specific medications, or a lack of proficiency in inserting an IV line, particularly among practitioners working outside the hospital settings, such as general practitioners. As further explained by some participants, in cases where IV access was unsuccessful, they were either required to switch to oral administration if they had previously ordered it as a backup or, alternatively, cancel the procedure and wait for the delivery of the oral medication from the pharmacy. Even though most cases were reported to have gone very smoothly, the thought of failing to facilitate the patient’s death on the chosen day and the subsequent disappointment experienced by both the patient and their family members weighed heavily on the minds of most participants, resulting in significant stress and emotional burden.

Malcolm: I think the only time it can get unpleasant is if you struggle to get an intravenous line in them. That has happened to me, you know. That makes it marginally stressful. Because you know the person wants to go. You want to facilitate the person to go. But you can’t get a line in, so yeah.

##### The performance of AD and the final moments: “I’d really like to be more of a background figure”

All participants described individualised and systematic approaches to patient care throughout the AD process. A shared element among these approaches was the prioritisation of patient and family-centred care, allowing space for patients and families, particularly during their final moments, to say farewell and engage in their preferred end-of-life rituals. Participants appeared to recognise the uniqueness of each patient’s experience, with some patients surrounded by numerous family members, holding a big farewell party, sharing memories and mixed emotions of tears and laughter, and even cracking jokes, while others chose to have their final moments alone or with a select few family and friends, with background music playing and in their favourite place. As one participant described the diversity of experiences:

Evan: Every patient is different. On their last day, some patients are quiet and pensive, thinking over the last 70-odd years of their life, while others want to hurry up and get it over with after so many months or years of suffering. The tone is set by the patient. It’s their life, their suffering, their day. It’s not about my experience.

Despite these differences, there was a prevailing sense of peace reported by ADPs and a “bizarre kind of calmness over what had been quite a stressful thing” during the final moments, with patients holding their loved ones’ hands and expressing gratitude before peacefully drifting away.

#### Perceived experience of patients and families with AD

##### End-of-life & AD literacy: “These people are sort of ahead of me in talking and thinking about it [death/AD]”

Some participants commented on how most patients they cared for were of Pākehā or European descent with a high level of education, and end-of-life literacy. These patients were perceived by participants as self-determined and autonomous individuals with fewer religious beliefs. According to participants, although the patients’ desire for AD was often consistent, they often encountered challenges in determining the appropriate channels to access the service. In addition, some participants noticed that patients appeared to have a limited understanding of the logistical aspects and duration of the AD process. ADPs identified several factors contributing to this situation, including societal discomfort in discussing death openly, insufficient public availability of accessible information about AD (especially for elderly individuals with limited computer literacy), and the statutory prohibition on healthcare providers initiating discussion about AD services.

##### Perceived impact of AD on patients and families: “There is a massive sense of relief and I’ve never not been thanked”

Most ADPs raised the concept of reclaiming control over a difficult and unsteady situation. They observed that patients appreciated the AD process as it provided them with relief and the ability to have some control over their own suffering and the process of dying. One participant described how patients’ quality of life improved through this control:

Malcolm: More than a few people have said that after I met them, and this is not necessarily because it’s me, but because they could find a finite point to their suffering. Their life improved considerably. In fact, even family members, after the events, say that once a date was set and she knew, or rather she knew, that their suffering was going to end, they brightened up. They were willing to see people, engage more, and it has been a very rewarding experience.

As reported by ADPs, families of patients requesting AD showed a combination of deep sadness due to losing a loved one, acceptance and gratitude that their loved one had the opportunity to die on their own terms. Participants noted that some patients were protective of their family members and chose not to involve them in decision-making, while others included them. In some cases, family members expressed disapproval of the AD choice and struggled to accept it, although this rarely became a significant issue. Participants also reported that despite any disagreements, families predominantly supported the dying patient and each other during the final moments and in the aftermath, with their primary concern being the patient’s well-being.

Some participants perceived aspects of the AD regulations and process led to patients and families feeling stress and frustration. These included delays and lengthy processes, particularly in acute conditions, the lack of cooperation and accommodation for AD in public hospitals, hospices, or home care facilities, the strict eligibility criteria, and instances where AD requests were denied.

##### Cultural safety & ADP’s experience with Māori who applied for AD: “The comments I have about Māori accessing end-of-life choice is they’re notable by their absence”

Māori are the indigenous people of New Zealand. Overall, our participants had limited interactions with Māori patients requesting AD. Some participants displayed hesitation and a lack of preparedness in discussing Māori end-of-life experiences during the assessment. These factors posed challenges in fully exploring cultural safety and the provision of culturally and spiritually sensitive care for Māori and their *whānau* (family) within the AD context.

Some participants demonstrated a flexible and responsive approach to meeting the needs of Māori patients and their whānau, recognising the importance of cultural differences and their impact on end-of-life care interactions. In contrast, some others held preconceived notions that Māori individuals would not seek AD if they identified strongly with their culture. This led to the exclusion of Māori cultural considerations from the AD process. However, participants primarily associated their understanding of Māori culture with the size of the whānau rather than considering the spiritual, ethical, and philosophical aspects in relation to end-of-life care.

##### Ensuring safety and family support: “I think you’d probably need more time to visit and give more information than is allowed under the current schedule for the Ministry of Health”

Some participants, particularly those residing in rural areas or providing services to culturally diverse communities, shared instances where patients had their extended family members present during assessment sessions. Most participants did not think that family members were pressuring patients to choose an assisted death., However, a few participants expressed concerns about potential coercion by family members to dissuade patients from pursuing an AD.

Another observation shared by participants was how quickly death occurred once IV medication had been administered, which often caught families off guard. Participants went on to highlight the importance of adequately preparing families beforehand. Moreover, participants described how they were sensitive to the diverse immediate grief reactions shown by families, thereby tailoring their responses accordingly. Some participants provided patients and families with their personal phone numbers, offering opportunities for families to seek clarification, engage in debriefing sessions, and address any concerns throughout the AD process, including the post-assisted death procedure. However, some participants described how they felt frustrated because they were unaware of the extent of professional support offered to families by the MoH following the completion of the AD process. Participants acknowledged that the grief process following an AD may differ from other forms of grief and reported that hospitals, in particular, typically did not offer bereavement support services. This increased their concerns about the lack of follow-up support for families.

### Theme 3: BEING – Being an ADP

#### Personal and professional impact of AD provision on ADPs: “Before an assisted death, I feel quite unsettled, I don’t sleep very well the night before”

Some participants expressed wrestling with clinical and legal uncertainties when first engaging in AD assessments, lacking a comprehensive understanding of their role and the process involved. For some, particularly prior to the administration of an AD, this uncertainty was reported to have transformed into fear and anxiety. An ethical and existential battle associated with the responsibility of intentionally ending someone’s life would occupy their thoughts. Some participants openly shared their fears with patients, seeking reassurance and validation of their actions. Engaging in challenging ethical conversations with themselves and trusted individuals was a common strategy to reconcile their involvement in the act of causing someone’s death, occasionally questioning whether it aligned with the principles they had taken an oath to uphold.

Some participants also reported anxiety, which stemmed from the desire to ensure a flawless and non-traumatic experience for families, facilitating a peaceful death as envisioned by the patient. Despite the ongoing emotional and intellectual challenges and the persistence of pre-administration anxiety, participants described how their confidence grew with each subsequent case. Indeed, most participants experienced a crucial shift in their mindset, transitioning from perceiving themselves as the sole agents responsible for ending a life to recognising their role as facilitators, enabling individuals to exercise their autonomy and fulfil their wishes legally. Furthermore, participants described how they experienced a profound sense of gratitude and honour following the administration process. All participants felt privileged and fulfilled by their involvement in a “deeply personal and sacred space,” where they could make a positive and meaningful impact by alleviating suffering and enabling individuals to have a dignified and peaceful death, hence supporting families in their grief.

Professionally, despite sometimes feeling isolated in their role, some participants expressed increased job satisfaction compared to their regular practice. They found that engaging in AD work allowed them to rediscover their long-held passion for helping people and re-establish a meaningful connection with their profession and patients as healthcare professionals. Meaningful encounters with those patients with AD requests prompted them to reflect on the essence of life and death and the importance of prioritising what matters to them. These experiences were reported to have reaffirmed their belief in respecting an individual’s autonomy to determine the manner in which they wish to live and die.

Chris: Positively. Again, being part of that, sort of, sacred, the end of somebody’s journey and watching and being in the space when someone passes away is a very special thing. And I think it makes me appreciate life more I think as well. Because I know I’ll be in that situation at some point in time. Hopefully, I can be looked after, but I guess being in the presence of death reminds you to enjoy life a little bit more. And, you know, tell your mum that you love her and all those things ‘cause things can change very suddenly. So, I think it’s made me even more appreciative of the job that I’ve got the work that I do and the life that I’ve got.

#### Impact of social stigma and secrecy: “I’m getting the impression that it’s still not really something you discuss. It’s almost something to keep secret”

Most participants described how patients expressed a desire for confidentiality. They described how some patients refrained from having a funeral or disclosing their choice of AD to their religious figures, GPs, community members, and sometimes even their own relatives. Participants perceived this need for secrecy as an indication of the shame and stigma surrounding an assisted death. Participants raised concerns about the potential for complicated grief among family members who may struggle to openly discuss the fact that their loved one opted for AD.

Some participants, particularly those from culturally diverse communities, reported facing resistance and negative judgment from their family and community members, who considered them to be a ‘bad omen’ that would bring misfortune upon the family or community. To avoid judgment, some participants described how they chose to keep their involvement in AD a secret.

Elena: I haven’t told anybody in my workplace that I’m doing this service. Because it’s a [Name] department and there’s quite a lot of Christian people there. And just philosophically, yeah, I’m not sure how it would go down.

In the workplace and throughout the wider community, the topic of AD was generally not openly discussed by ADPs and the patients they cared for. Some participants expressed concerns about damaging their relationships and facing judgement from conscientious objectors, leading them to keep their involvement with AD hidden from their colleagues. They described how secrecy not only contributed to a lack of support and feeling isolated but also hindered educational learning for practitioners and the normalisation of assisted death within society. Furthermore, concerns regarding the associated stigma prevented some participants from accepting AD referrals from within their communities, as they hoped to preserve their anonymity within the community and maintain intact relationships with the people involved. There was a sense of fear among ADPs, particularly those in rural areas, that their involvement in an assisted death would eventually become public knowledge. Conversely, some were open about their participation but expressed anxiety about encountering conscientious objectors who may confront them with objections and criticism.

#### Emotional wellbeing and clinical safety of ADPs: “You have to be able to ventilate a little bit and to reflect, and to take care of yourself”

Most participants in this study reported that their involvement in AD provision did not result in significant psychological distress or trauma, as the positive aspects of their work outweighed the associated stress. However, they acknowledged that it was still an emotionally charged experience that sometimes evoked tears or deep empathy for the patients. To maintain a healthy balance and minimise the impact, participants employed various self-care strategies. The most common coping strategies were taking the rest of the day off and engaging in debriefing sessions with colleagues, supervisors, family members, or friends. Additionally, some participants reframed their perception of AD by treating it as a spectrum of good palliative care, aiming to mitigate the negative emotions associated with the practice.

Participants highlighted the presence of a colleague during the AD administration, and their availability for professional, clinical, and emotional support as a proactive intervention method. This support was highly valued, especially during the initial AD experience, and it was preferred that the colleague be familiar with the procedure. Engaging in conversations about AD, sharing emotions with peers, and having professional supervision played a significant role in promoting the mental well-being of participants who had access to such support.

Andre: You do feel very alone doing the job. Unless you happen to be working with other people who are doing it. But as I said, because there’s sort of a fight club scenario, you don’t talk about it.

However, some participants mentioned the unavailability of peer support after their first AD administration. This was despite the availability of funds for a support practitioner. Participants described how busy schedules or a lack of interest in involvement with AD prevented them from identifying a support practitioner. Having a support practitioner present on-site during the AD was also seen as a safety measure for the ADP:

Jared: My main concern is that if a relative made an allegation about me, I would have no witnesses. Nobody to support me. You know, the only other witness I’ve just helped to die.

#### Balancing the load: “I am conscious about not having too many in quick succession and spacing them out”

Participants highlighted the significant demands on most ADPs, who often need to clear their schedules, take time off work, or conduct assessments outside regular working hours. This is especially challenging for ADPs traveling long distances in rural areas. Financial remuneration for their time and effort was reported insufficient compared to alternative work, presenting a financial obstacle. Additionally, ADPs face the burden of investing in professional development without full reimbursement, particularly those not affiliated with public health services. Inadequate financial software systems complicate managing and tracking reimbursements. In their commitment to ensuring quality care and comprehensive assessments, some participants mentioned going beyond the measured norm for reimbursement. Despite their dedication, participants voiced concerns about the escalating workload and demand for AD services, which may force them to decline referrals or limit their roles, potentially impacting their continued involvement.

Participants indicated that the professionalism and promptness of the AD secretariat and clinical advisory team had made being an ADP easier. Participants expressed widespread satisfaction with the support from the MoH’s AD service: “The three clinical advisers I’ve worked with have ranged from good to exceptional in every interaction and were always helpful.” The support covered case coordination, information provision, guidance on diverse topics, assistance with applications and paperwork, IV access issues, and facilitating debriefing sessions. Participants particularly depended on the advisory team for navigating the electronic portal for AD applications. Initially, they faced challenges with uploading materials, organizing forms, and accessing notes from independent medical practitioners. These challenges seem to have eased with experience. Additionally, participants valued access to patients’ hospital/medical records during assessments, though not all ADPs had this access.

The regulatory prohibition on raising AD, the workforce-related challenges, and delays imposed by, for example, conscientious objectors and other mentioned obstacles have raised concerns regarding the timely provision of AD services. Several participants expressed frustration with the perceived inability to expedite the process, especially in rapidly progressive conditions. Conversely, some participants viewed this aspect as a deliberate feature serving the purpose of a “cooling-off period” that allows patients and their family members to reflect and prepare before proceeding with the AD service.

## Discussion

This study is among the first to explore the experiences of practitioners directly involved in providing AD services in Aotearoa New Zealand. The findings present an overview of AD service provision experience from the perspectives of 22 ADPs. We present an overview of their journey from theory to practice: from ‘knowing’ about AD only theoretically to ‘doing’ an AD provision and ‘becoming/being’ an ADP. While we discuss these themes distinctly, it is important to note that they are intertwined, reflecting a non-linear progression in the ADPs’ experiences, and are influenced by other overarching factors such as health system infrastructure, challenges, and resources available. For example, for ADPs in New Zealand, the practice of AD is largely an individual/solo practice, unlike other countries where AD is usually practised in a team [30]. This has created unique characteristics that impact some aspects of the ADPs’ experience. Despite being novice AD practitioners, most ADPs who participated in this study were motivated to take on the challenges of undertaking a complex task that is emotionally charged and carries some degree of stigma in society and the medical community. Practitioners who are new to AD would benefit from learning about the rich collective experience reported in this study [31].

*Knowing About and Doing AD*. For Heidegger (2010), knowledge and feelings are interconnected in learning, and they shape two forms of knowing: *Erkennen* (knowing) and *Verstehen* (understanding). Erkennen represents the traditional sense of theoretical-practical knowledge, while Verstehen is closely related to the concepts of interpretation and the revelation of hidden meanings [29]. Heidegger (2010) believed that the personal, cultural, and social aspects of individuals’ existence and experiences shape their perspective, interpretation, and experience of reality [29].

In this study, the reality is the experience of providing AD. As also experienced by participants in this study, literature shows that practitioners in various countries face challenges due to the lack of clarity in policies governing AD. These policies often require individual interpretation by practitioners [5, 32]. This means that practitioners may incorporate their personal perceptions, values, and understanding of the criteria, legal procedures, and the situation at hand, which may lead to responses beyond the formal policy. For instance, as also found in this study, the unbearable suffering criterion is likely to prompt individual interpretation influenced by personal values [33]. Some of the most reported personal and professional values that impact response to AD requests are relieving patients’ suffering, empowering patients’ right to autonomy and their families, and enhancing their end-of-life quality [34, 35].

As expressed by participants in this study, obstruction of access to AD service or delay in its delivery may occur if other practitioners involved in the patient’s end-of-life care and AD assessment process, such as GPs and palliative care practitioners, do not share the same values and/or understanding of that process. Differences in practitioners’ opinions, understanding, and experiences regarding AD have been established in the literature [6, 36, 37]. Research on how these personal differences impact AD service delivery on stakeholders is also growing [8, 11, 32, 38–41].

A recent study involving interviews with nurses in Canada revealed that they were deeply troubled by certain palliative care practitioners whom they believed had obstructed patients’ access to AD [39]. This obstruction included disregarding patient requests or discontinuing palliative care services after a decision for AD had been made [39]. Findings from this study suggest obstruction can be due to individual and institutional objections to AD. In New Zealand, most regulatory bodies, including most hospices, follow the conscientious absolutism model that permits institutions to object to providing AD without limit [42]. The application of the conscientious absolutism model, coupled with the reported findings of obstructive behaviour due to individual conscientious objection, has delayed and restricted access to AD and further added to the ADPs’ workload. Some examples include either deliberately or sub-consciously communicating misinformation or withholding information about AD from patients; additional attempts by ADPs to find alternative ways to elicit diagnostic information when the patient’s doctor refrains from conveying this information due to being an objector; and difficulty finding AD delivery sites when community facilities such as hospices or aged residential cares won’t allow AD to be delivered on their premises. These findings are also supported by recent research on healthcare professionals’ experiences in New Zealand [11].

Limitations on the ability to interpret policies, eligibility criteria and differences in core values that facilitate and provide AD services indicate that there is a need for practitioners to acquire further knowledge and adapt their skills to provide this service. This is essential, particularly in countries with newer established policies.

During early AD implementation, the newly becoming ADPs have limited theoretical/practical knowledge and encounters with actual AD experiences. In addition, the nature of incurable life-limiting illnesses and end-of-life care itself, calls for multidisciplinary theoretical and practical knowledge [43, 44]. Consistent with findings from this study, other international studies have reported instances where ADPs were confronted with unexpected challenges and situations where they had little knowledge and/or control over. Some examples include difficulty assessing some of the eligibility criteria, such as making an accurate prognosis [32, 45, 46]; sudden changes in disease trajectory before the administrative process is completed, leading to rushed decisions to bring the AD date forward or expediting the process [8]; oral medication causing prolonged death with unexpected reflexes such as vomiting or gagging [8, 30, 47, 48]; and difficulty getting an IV access for administering medication [8, 48]. Overcoming these complications for new ADPs could be clinically and emotionally challenging. With the collective knowledge and support of a colleague or a team, these challenges could be better addressed during the AD assessment or administration process, minimising the risk of drawbacks and emotional and technical surprise, hence diminishing the risk of their implications on patients’ and ADPs’ wellbeing. The MoH has been collecting feedback and implementing measures to tackle challenges in AD practices. For instance, the introduction of intraosseous injection was implemented after reports of ADPs having difficulties with IV access in some patients. However, there is a need to study further and adapt the AD service using the early experiences of ADPs. Adjustments are essential as experiences with the AD access and process unfold.

*Being an ADP.* Findings from this study align with international research, which highlights risks with any new treatments or procedures [49]. Apart from practical issues of adhering to the AD process, arguably the most critical adverse outcome is the inappropriate death of the person and the impact of this on others, including ADPs and families. A recent literature review argues that there is a notable lack of empirical evidence on AD to improve end-of-life care outcomes for individuals with terminal illnesses and their families [49]. In addition, numerous studies have indicated the potential negative impacts on practitioners involved in AD provision, including emotional and moral distress, as well as the adverse consequences of communal and societal stigma [11, 36]. These factors often compel some practitioners to conceal their involvement, underscoring the necessity of fully comprehending these implications. A question that arises, therefore, is what balances the experience of AD against its risks for providers, motivating them to continue providing AD.

Based on the phenomenological viewpoint on the concept of experience, as reflected in the work of Van Manen (1977), experience is future-oriented and requires openness and choice. It is the experience of reflection that contributes to our knowledge and comprehension of a subject. In this sense, “experience refers to the phenomenon of the immediacy with which something real is grasped, in contrast to something one presumes to know, but the confirmation of which comes through one’s own experience” [29]. Through this study, ADPs were engaged in a continual process of abstraction and reflection, which led to a shift in their presumed perception of AD. The initial uncertainty and moral dilemmas they faced gradually yielded to the emergence of more positive outcomes associated with their involvement in AD. Some of these positive outcomes, as depicted by our findings and existing research, are a personal sense of fulfilment in helping and empowering others, having a meaningful impact on someone else’s life by alleviating suffering, and receiving validation from families based on improved quality of their loved one’s end-of-life experience [8, 30]. These gratifying aspects of ADPs’ experience were often used to rationalise their part in someone else’s death, respond to the existential dilemmas associated with AD, and cope with any internal challenges.

Recognising and exploring this cognitive dissonance, described by some ADPs, between their expectation and actual experiences is crucial to developing best practices in this emergent field. Further research into the specific aspects and instances causing this dissonance and designing and implementing programme, including both technical skills and emotional resilience strategies, that prepare ADPs for AD may advance our understanding and practice in the AD field. The MoH regulates the AD service, and as of March 2023, the operational responsibilities shifted to Health New Zealand [50]. These governmental bodies have extended support to ADPs by setting up regional AD peer network groups throughout New Zealand. Although the nature and extent of this support are adapting to the increasing demand for AD services, ADPs in each region have autonomously organised informal peer groups. This trend suggests that the support system is developing and becoming more established over time. ADPs discovered value in both formal and informal peer groups, yet they expressed a greater preference for the informal ones. The benefits, according to most ADPs, were the opportunities to connect with other ADPs in the same area, engage in discussions about cases and challenges within a safe environment, seek guidance, and share resources, thus facilitating AD provisions. These peer support groups have also been highly regarded in another study conducted in New Zealand [11].

In this study, we identified some external barriers that may influence the intention of the continuum of AD provision. Systemic issues impacted the quality of AD provision. These issues are consistent with local and international literature [11, 51, 52]. Barriers to AD are related to challenges facing the current AD workforce, including their work schedule and workload. Additional barriers include the statutory prohibition that prevents healthcare providers from initiating AD discussions and its potential impacts. Finally, there are barriers related to remuneration and having responsibilities beyond standard reimbursement and loss of income, which can become a challenge, especially as the demand for AD increases [53]. Overall, the combination of time constraints, inadequate remuneration, and additional unpaid responsibilities poses obstacles for ADPs in providing AD services. Addressing these challenges is crucial to ensure the sustainability and accessibility of quality care in this field.

Equitable access and delivery of AD services for Māori require more attention. With little known about Māori people’s needs, beliefs, and protocols regarding AD [54], research is currently underway to gain greater insight into the meaning of AD for Māori people and how culturally centred and safe care can be provided. Findings from this study indicated issues with cultural safety and competence, suggesting that a greater effort must be made to ensure ADPs incorporate cultural care customs in their approach to processes involved with carrying out an assisted death. Furthermore, findings revealed that participants did not differentiate between cultural considerations for end-of-life care and AD, despite the potential differing implications for patients and their whānau. An example of implication would be the story of a Māori patient, shared by a participant, who concealed their application for AD, expressing a concern about guilt and a fear of judgment from the cultural community. Our findings also suggest current care is provided based on ADPs’ previous experience with Māori and is heavily influenced by stereotypical assumptions. The power differential with AD services must not be disregarded as a barrier for Māori to access AD services [55].

## Conclusion

The findings of this study illustrate the interplay between clinical specialisation, practice settings, institutional and individual conscientious objections, legal, and societal challenges, and the motivation to embrace the role of ADPs and the continuum of AD provisions against the odds. Some of the challenges are the shared instances of what is described by the Canadian study [18] as a ‘failure of imagination’, where ADPs encountered adverse situations that, in retrospect, appear foreseeable, but were not adequately anticipated during the implementation phase. Additionally, the geographical distribution of ADPs, which correlates with existing healthcare disparities in New Zealand, further shapes the landscape of AD services. In this study, we also found that the discrepancy between the timeframe requirement and the actual progression of certain illnesses creates tension and raises important considerations for the eligibility criteria in AD cases. These findings shed light on the perceived strictness of eligibility criteria for AD and the practical challenges encountered during the assessment process. The assessment of eligibility criteria was also challenged by ambiguity in the definition of some criteria, such as unbearable suffering and terminal illnesses and their applications. Discussing the unspeakable, the act of dying or providing an assisted death that has not yet become a societal norm, and the absence of cultural and societal understanding around it, continues to influence the experiences of patients, families, and practitioners with AD. Understanding these perspectives and experiences is crucial for ongoing discussions and potential revisions to ensure a more balanced and patient-centred approach to providing AD practices.

### Limitations and recommendations

It is important to acknowledge that the viewpoint predominantly expressed by most ADPs was around the perception that AD eligibility criteria, although important, are too strict. This may introduce a potential limitation regarding the generalisability of findings to non-ADPs, as ADPs are likely to hold pro-AD views and are biased by their own personal view on AD. Another limitation to consider is the composition of our participant groups, which displayed a higher representation of European New Zealanders individuals and a lower representation from rural areas. A similar pattern of diversity was observed among the patients that ADPs were involved with, with most cases involving European New Zealanders individuals. While this distribution somewhat mirrors the diversity of ADPs and patients using AD services in New Zealand, it raises the question of whether the perspectives of Māori practitioners and views on Māori end-of-life culture in the context of AD might be underrepresented or not fully captured. On a positive note, it is worth mentioning that there are currently parallel studies underway specifically focused on capturing the experiences of Māori patients and families involved in the AD process. These ongoing studies aim to provide a more comprehensive understanding of the AD experience within the Māori community, which will complement and enrich the existing body of knowledge. Another noteworthy limitation of this study is that while ADPs play a crucial role in the AD process and interact closely with families and patients, the findings are based on the ADPs’ interpretations of these experiences rather than direct accounts from the families and patients themselves. Further research is recommended to capture the full nuances and complexities of the actual experience of patients and their families.

## Electronic supplementary material

Below is the link to the electronic supplementary material.

Supplementary Material 1

## Data Availability

The datasets generated and/or analysed during the current study are not publicly available due to confidentially concerns but are available from the corresponding author on reasonable request.

## Abbreviations

AD: Assisted dying
ADP: Assisted dying practitioner
AMP: Attending medical practitioner
ANP: Attending nurse practitioner
IMP: Independent medical practitioner
The Act: End of Life Choice Act 2019
MoH: Ministry of Health
GP: General practitioner
IV: Intravenous
