# Supplementary material for: Early experiences of the End of Life Choice Act 2019 amongst assisted dying practitioners in Aotearoa New Zealand
Source: BMC Palliat Care. 2025 May 24;24:149. doi: 10.1186/s12904-025-01747-w (PMC12102867; doi:10.1186/s12904-025-01747-w)
Supplement: Supplementary file 1 — Supplementary Material 1 [file 12904_2025_1747_MOESM1_ESM.docx]

**Semi-structured interview – Early experience of AD practitioners with the EoLC Act**

**Direct involvement**

1. You mentioned earlier that you have been directly involved with a patient who requested assisted dying. **Can you please describe your experience?**
2. Can you describe your role as a health practitioner?
   1. Did you have a prior relationship with the patient?
   2. How did it help / not help provide the assisted dying service?

**Eligibility Requirements**

Now I’d like to focus on the eligibility requirements for assisted dying.

1. Could you tell me how you find the eligibility criteria from the Act?
   1. Are there any specific criteria you would like to comment?
   2. Could you tell me about your experience applying these criteria? Prompts: how did you know what they were/what’s working /not working and why?
   3. How did you find assessing the patient’s decision-making capacity?
      1. How did you overcome any potential uncertainty?
      2. What tools did you use?
   4. How did you find the assessment of suffering? Prompts: physical, emotional, mental, existential, etc.
   5. What is your view on the AD process timeline? Advantages/disadvantages etc.
   6. What is your view on the fact that, as a health practitioner, you are not legally allowed to initiate discussion about AD as one of their end-of-life options?
   7. How did you manage a case who was found not eligible (if any!)?
      1. Would you be worried about them being willing to take their own life because of not being eligible?
   8. Are you aware of any cases where patients/families request AD but were given misinformation or abandoned and not referred to someone who provides such a service?

**Administration**

1. Have you been involved in administrating the medication on the day of an assisted death? Have you been present on the day of an assisted dying death? If yes,
   1. How is this working in practice? What’s helping / not helping?
   2. How did you find using the medication kit?
   3. How did you find the final check of competency to make an informed decision?
   4. What was it like for you being there?

**Whānau/Family**

1. Is there anything that stood out in your interactions with the whānau/family before, during, or after the procedure (i.e., any memorable comments)? Prompt: patient's emotions, family’s emotions, your emotion?
2. Based on your experience(s), do you think anything can be improved regarding whānau/family experience? Prompt: transportation issues, location/setting, team composition.

**Working with Māori**

1. Have you worked with a Māori patient who requested assisted dying?

If yes,

1. Can you please describe your experience?
2. How was your interaction with the patient’s whānau?
3. Can you describe how you negotiated your role as a Māori/non-Māori health professional and working with Māori patients and/or their whānau in assisted dying?
4. How was Tikanga followed or incorporated into assisted dying?
5. How did you look after your Wairau? (For Māori HPs)
6. How did you look after yourself regarding cultural safety? (For both Māori and non-Māori HPs)
   1. What are some of the discomforts you may have with the process?

**Resources**

1. How did you find help and support from the MoH?
2. What resources did you use in the process?
3. Are you aware of any EoLC informal supports e.g., peer review groups or online forums available for health practitioners? If yes,
   1. Are you involved in any of them?
4. What changes do you think are required to improve the provision of assisted dying?
   1. What additional learning or training would be of help to you?

**Self-Care**

1. How do you feel your involvement in assisted dying has had any impact on you personally and/or professionally? Prompts: relationship with your colleagues, patients, your family, and friends?
2. How do you look after yourself?
3. How do your peers look after you? Or how much support do you receive from your colleagues?
4. Did you encounter any ethical or moral complexity/dilemma? If yes,
   1. can you describe that? How did you resolve the dilemma?
5. Considering your experience, how much are you willing to provide assisted dying in the future?

**Last comment**

1. Is there something else that you would like to add before we finish the interview?
